# Multiple Independent Fusions of Glucose-6-Phosphate Dehydrogenase with Enzymes in the Pentose Phosphate Pathway

**DOI:** 10.1371/journal.pone.0022269

**Published:** 2011-08-01

**Authors:** Nicholas A. Stover, Thomas A. Dixon, Andre R. O. Cavalcanti

**Affiliations:** 1 Biology Department, Bradley University, Peoria, Illinois, United States of America; 2 Chemistry Department, Pomona College, Claremont, California, United States of America; 3 Biology Department, Pomona College, Claremont, California, United States of America; University of Georgia, United States of America

## Abstract

Fusions of the first two enzymes in the pentose phosphate pathway, glucose-6-phosphate dehydrogenase (G6PD) and 6-phosphogluconolactonase (6PGL), have been previously described in two distant clades, chordates and species of the malarial parasite *Plasmodium*. We have analyzed genome and expressed sequence data from a variety of organisms to identify the origins of these gene fusion events. Based on the orientation of the domains and range of species in which homologs can be found, the fusions appear to have occurred independently, near the base of the metazoan and apicomplexan lineages. Only one of the two metazoan paralogs of G6PD is fused, showing that the fusion occurred after a duplication event, which we have traced back to an ancestor of choanoflagellates and metazoans. The *Plasmodium* genes are known to contain a functionally important insertion that is not seen in the other apicomplexan fusions, highlighting this as a unique characteristic of this group. Surprisingly, our search revealed two additional fusion events, one that combined 6PGL and G6PD in an ancestor of the protozoan parasites *Trichomonas* and *Giardia*, and another fusing G6PD with phosphogluconate dehydrogenase (6PGD) in a species of diatoms. This study extends the range of species known to contain fusions in the pentose phosphate pathway to many new medically and economically important organisms.

## Introduction

Glucose-6-phosphate dehydrogenase (G6PD) is the first enzyme of the pentose phosphate pathway, a conserved pathway responsible for producing a variety of fundamental molecules, including nucleotide precursors and NADPH [1]. NADPH is an important source of electrons used in many cellular reactions, particularly by enzymes involved in the regulation of oxidative stress. Accordingly, this co-factor is involved in at least three antioxidant pathways, the glutathione, thioredoxin, and glutaredoxin cycles. In the glutathione cycle, NADPH regenerates the free radical scavenging molecule glutathione (GSH) after its oxidation to glutathione disulfide (GSSG). Electrons from NADPH are donated during the reaction catalyzed by glutathione reductase, in which GSSG is split to release two molecules of GSH [2]. NADPH performs a similar role in the other antioxidant pathways.

**Figure 1:**
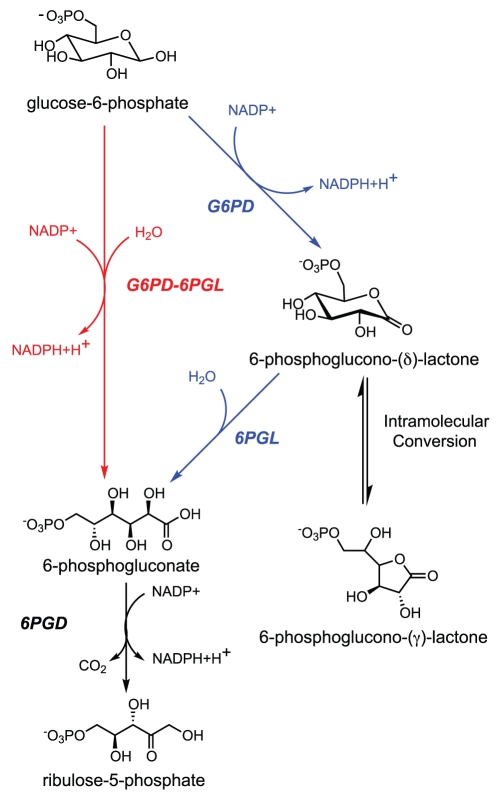
The oxidative branch of the pentose phosphate pathway. The route taken by organisms with bifunctional enzymes is shown in red, while the route taken by other organisms with a functioning pentose phosphate pathway is shown in blue.

Figure 1 shows the initial steps of the pentose phosphate pathway, which constitute the oxidative phase of the pathway [1]. After hexokinase (EC: 2.7.1.1) phosphorylates glucose upon its entry to the cell, glucose-6-phosphate-1-dehydrogenase (G6PD; EC: 1.1.1.49) catalyzes its conversion to 6-phosphoglucono-(δ)-lactone. A molecule of NADPH is produced as a result of this reaction. Next, 6-phosphogluconolactonase (6PGL; EC: 3.1.1.31) converts this product into 6-phosphogluconate. A second molecule of NADPH is produced as this is converted by 6-phosphogluconate dehydrogenase (6PGD; EC: 1.1.1.44) into ribulose-5-phosphate.

Although 6-phosphoglucono-(δ)-lactone, a δ-lactone, can undergo spontaneous hydrolysis to form 6-phosphogluconate, 6PGL is required for efficient functioning of the pathway. In the absence of 6PGL, the δ-lactone will undergo a conversion to the γ-lactone form, which does not undergo spontaneous hydrolysis and builds to toxic levels in the cells. The 6PGL enzyme thus ensures that the δ-lactone product of G6PD will be hydrolyzed rapidly, before its conversion to the γ-lactone [1].

Vertebrates have two versions of the oxidative phase of the pentose phosphate pathway. In addition to G6PD, these organisms possess an enzyme called H6PD (hexose-6-phosphate dehydrogenase) that can catalyze the same reaction as G6PD. H6PD, also referred to as glucose dehydrogenase (GDH), has extensive sequence similarity to G6PD and is believed to have been derived from this enzyme by sequence divergence following a duplication event [3]–[8]. Despite their similarities and common origin, many functional differences have been reported between the two enzymes. Biochemical studies have shown that H6PD has lost specificity for glucose-6-phosphate as its sole substrate and is able to oxidize additional hexose-6-phosphates (e.g. galactose-6-phosphate, glucosamine-6-phosphate) as well as simple monosaccharides like glucose [9]. H6PD also uses multiple nucleotide co-factors and can produce both NADPH and NADH. A unique function for H6PD has also been identified: the generation of NADPH for use by 11 β-hydroxysteroid dehydrogenase (11 β-HSD1) in the activation of glucocorticoids in the ER [9]. Differences in the cellular and tissue localization of these two proteins have also been observed. G6PD is a cytoplasmic enzyme found in a wide range of tissues [9], [10], whereas H6PD is limited to microsomes and is notably absent in human red blood cells [11].

The H6PD protein sequence extends beyond that of G6PD, and it has been shown that the C-terminus of this enzyme has 6PGL activity. The source of this additional sequence has been identified as a gene fusion event that produced a chimeric protein capable of catalyzing the first two steps of the pentose phosphate pathway [8], [12]. Interestingly, another fusion protein containing these two genes has been described in species of *Plasmodium*. However, the orientation of the metazoan and *Plasmodium* genes is different, with 6PGL found at the N-terminus of the *Plasmodium* proteins. This difference in orientation indicates that the G6PD and 6PGL genes fused independently in these two lineages. In order to determine when these gene fusions occurred and to identify what other species are likely to have similar fusions, we have undertaken a search for the evolutionary origins of these 6PGL/G6PD fusion events. This search quickly led to the identification of a third independent fusion of these genes in the lineage of two distantly related eukaryotic parasites, *Giardia lamblia* and *Trichomonas vaginalis*. A fusion of G6PD with 6PGD, another enzyme of the pentose phosphate pathway, was also discovered in the diatom *Phaeodactylum tricornutum*. The implications of these fusions and their distributions are discussed.

## Methods

### Data Sources

To get a complete picture of the distribution of G6PD in eukaryotes we surveyed genome data from several data sources. In addition to all protist genomes available at the NCBI website (http://www.ncbi.nlm.nih.gov/sutils/blast_table.cgi?taxid=Protozoa), we searched all protist genomes currently available at EuPathDB [13] and at the DOE Joint Genome Institute (JGI; http://www.jgi.doe.gov/), which specialize in parasitic protozoa and algae, respectively. In addition to protist genomes we also included in our searches all fungal genomes in EuPathDB and a selection of fungal, metazoan and plant genomes available from GenBank. Finally, genome data for some organisms of phylogenetic importance to this study are only available at individual websites: the red algae *Cyanidioschyzon merolae* (http://merolae.biol.s.u-tokyo.ac.jp/) and *Galdieria sulphuraria* (http://genomics.msu.edu/galdieria/); the apicomplexan *Eimeria tenella* from the Sanger Center (ftp://ftp.sanger.ac.uk/pub/pathogens/Eimeria/tenella/); and the sponge *Amphimedon queenslandica* (http://spongezome.metazome.net/). Searches of EST databases were performed for additional taxa as needed (see Results).

### Identification of 6PGL/G6PD fusions in additional apicomplexans

The *Saccharomyces cerevisiae* G6PD enzyme Zwf1p (NCBI: NP_014158) was identified at SGD [14] and used to search the genomes listed above using BLASTP [15]. Detailed analysis of the BLAST results led to the discovery of 6PGL/G6PD fusions in all available apicomplexan genera except *Cryptosporidium*. TBLASTN searches using both Zwf1p and Sol1p (*S. cerevisiae* 6PGL homolog, NCBI: NP_014432), as well as the homologous proteins identified in other apicomplexans, were performed against the *Cryptosporidium* genome sequences to verify that the gene was not missed during gene model annotation. *Eimeria tenella* gene predictions were downloaded from the Sanger Center FTP site (ftp://ftp.sanger.ac.uk/pub/pathogens/Eimeria/tenella/) and searched for homologs of Zwf1p. The gene identified (TWINSCAN_PHASES00000241296) was further analyzed and discovered to be a fusion.

The searches described above revealed a G6PD homolog in the *Giardia lamblia* genome. The 2007 *G. lamblia* genome paper stated that no 6PGL gene was present in the genome [16], and the gene we identified was listed only as G6PD. However, closer inspection showed this to be a fusion of the two genes in the opposite orientation (G6PD/6PGL). The presence of 6PGL as part of this gene was undoubtedly overlooked due to the greater size and level of sequence conservation of the G6PD portion of the gene. After recognizing G6PD/6PGL as a possible arrangement of these two genes, we revisited genomes of the other species analyzed and discovered three homologous fused genes in *Trichomonas vaginalis* [17]. The *Trichomonas* genes were also annotated only as G6PD. A fusion between G6PD and 6PGD was found in the diatom *Phaeodactylum tricornutum* [18]. Except for the metazoan H6PD fusions described below, none of the other species analyzed appeared to contain fusions involving homologs of the G6PD gene.

In order to determine if any species not included in our survey of fully sequenced genomes contain G6PD/6PGL fusions, we performed BLASTP searches against the non-redundant (nr) database of proteins at GenBank. For these searches we used the full-length fusion proteins identified from several species to search for protein hits that spanned the entire length of the query. Special attention was paid to other alveolate species, which include ciliates and dinoflagellates in addition to the apicomplexans, and to the excavates, the clade that includes both *Giardia* and *Trichomonas*.

### Search for H6PD fusions in eukaryotes

We used the human H6PD protein sequence to search for putative orthologs in all the analyzed genomes. H6PD is derived from and shares sequence similarity with G6PD, but despite their common origin, the H6PD and G6PD sequences are sufficiently diverged that their genes are easy to differentiate using BLAST. If the best hits to an identified homolog were primarily to G6PD, we classified the hit as an ortholog of G6PD; if the best hits to the identified homolog were primarily to H6PD, we classified it as an ortholog to H6PD. The presence of additional domains was determined for each putative protein using the Conserved Domain search option at NCBI [19].

### Phylogenetic analyses

To test different evolutionary scenarios for the emergence of the fusion genes described here, we built phylogenetic trees using the G6PD domains of genes from a variety of species. For each set of sequences we used the CD (conserved domain) search feature of the NCBI BLAST webpage to determine the location of the G6PD domains [19]. We used only the parts of the sequences corresponding to these domains to build alignments. All analyses were performed using SeaView [20]. The sequences were aligned using MUSCLE [21] with default parameters.

For the maximum likelihood analysis we first used the program ProtTest [22] to select the amino acid substitution model that best fits the protein alignments. In both datasets, ProtTest determined that the model of amino acid substitution that best fit the data was the LG model [23], with invariable sites and across site rate variation. We used PhyML 3.0 [24], as implemented in SeaView to create maximum likelihood trees using the LG model with optimized number of invariable sites and optimized across site rate variation. Bootstrap support was calculated using the aLRT model [25]. The trees were visualized and prepared for publication using FigTree (http://tree.bio.ed.ac.uk/software/figtree/).

## Results

### Distribution of G6PD and H6PD in eukaryotes

**Figure 2:**
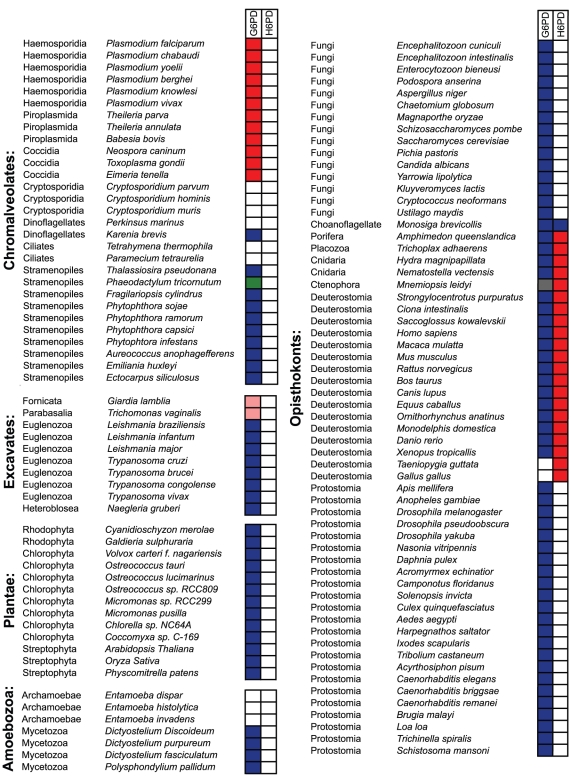
Species used in this study and their G6PD homologs. The species listed are divided into the major eukaryotic supergroups and subtaxa mentioned in the text. The Rhizaria is absent due to the lack of a fully sequenced genome from this supergroup. The two boxes show the fusion state of the G6PD and H6PD orthologs in each species. Red boxes represent a G6PD or H6PD at the N-terminus and 6PGL at the C-terminus; pink boxes represent a fusion with 6PGL at the N-terminus and G6PD at the C-terminus; blue boxes indicate the presence of a free, non-fused G6PD; the green box represents a fusion between G6PD and 6PGD; white boxes indicates that the species lacks a G6PD or H6PD ortholog. The grey box by the ctenophoran *Mnemiopsis leidyi* indicates that we could not identify a G6PD ortholog in this species, but, because we used only EST sequences, we cannot determine if G6PD is absent. Table S1 lists the accession numbers for all G6PD and H6PD orthologs shown in the figure.

Figure 2 shows the 119 eukaryotic species used in this study. The presence or absence of G6PD and H6PD genes is noted for each species, as is the fusion state of these genes (either fused or non-fused). Table S1 lists the source database for each organism (GenBank, JGI, EuPathDB, or individual genome databases) and accession numbers for all sequences analyzed. Since negative search results derived from an incomplete genome would be irrelevant, we restricted our searches to fully sequenced genomes. However, for large groups with no representative genome, or a genome that lacked homologs of G6PD or H6PD, we expanded the search to all proteins in GenBank and to EST sequences. For the dinoflagellate *Karenia brevis* the use of EST data allowed the identification of a G6PD homolog, and EST sequences from the Ctenophore *Mnemiopsis leidyi* allowed the identification of a fused H6PD gene in this species. One sequence, an EST from the ciliate *Ichthyophthirius multifiliis*, was removed from the analysis based on its absence in the completed genome sequence and its similarity to bacterial G6PD. Symbiotic bacteria are common in this species, and the EST may represent a contaminant from one of these cells. In addition to these directed searches of key species, we also searched the nr database at NCBI to determine if any partially sequenced species contained fused G6PD genes. These searches were all negative.

Current phylogenies tentatively divide the eukaryotes into six major groups [26], although there is some controversy regarding the monophyly of some of these groups [27], [28]: Plantae, composed of red algae, green algae and streptophytes; Chromalveolata, composed of alveolates and stramenopiles; Rhizaria, a little studied group that includes cercomonads, euglyphids and foraminiferans; Amoebozoa, composed of lobose amoebae and slime molds; Opisthokonta, composed of metazoans, fungi and choanoflagellates; and Excavata, a diverse group including fornicata, euglenozoans and parabasilids. Our dataset included genomes (or sequences derived from EST projects) from five of the six major eukaryotic groups: 29 Chromalveolata (2 ciliates, 2 dinoflagellates, 15 apicomplexans and 10 stramenopiles), 10 Excavata (1 fornicata, 1 parabasalid and 8 euglenozoa), 7 Amoebozoa, 13 Plantae (2 red algae, 8 green algae and 3 viridiplantae) and 60 Opisthokonta (15 fungi, 44 metazoans and 1 choanoflagellate) (Figure 2). Our searches turned up independent fusions of G6PD and 6PGL in three of these six supergroups. Unfortunately, no Rhizaria genome is currently available and EST searches did not return a G6PD homolog in this group.

### G6PD/6PGL fusions in alveolates and excavates

**Figure 3:**
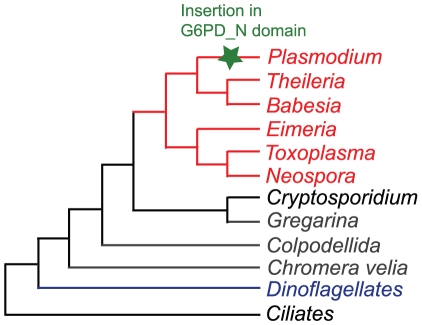
Phylogenetic tree of alveolates. Adapted from [36]–[38]. The taxa in red contain a fused version of G6PD; taxa in blue contain a non-fused gene. The taxa in black lack an ortholog of G6PD, while those in gray have no fully sequenced genome and have no G6PD sequence available for analysis. The green star in the *Plasmodium* branch of the tree represents the insertion observed in all *Plasmodium* species.

G6PD and 6PGL are fused in all of the currently available apicomplexan genomes with the exception of those of *Cryptosporidium spp.* (*C. parvum, C. homini, and C. muris*), in which both genes are absent. Extending the searches to the closest relatives of apicomplexans, the dinoflagellates, revealed that the genes are absent in the only fully sequenced organism in this group, *Perkinsus marinus*, although an expressed sequence tag (EST) encoding G6PD has been sequenced in the dinoflagellate *Karenia brevis* (NCBI: CO062433 and CO064937). Dinoflagellates and apicomplexans, along with ciliates, form the monophyletic group Alveolata (Figure 3). Two ciliate genomes have been fully sequenced, *Tetrahymena thermophila* [29] and *Paramecium tetraurelia* [30], neither of which contain fused or non-fused copies of G6PD or 6PGL.

**Figure 4:**
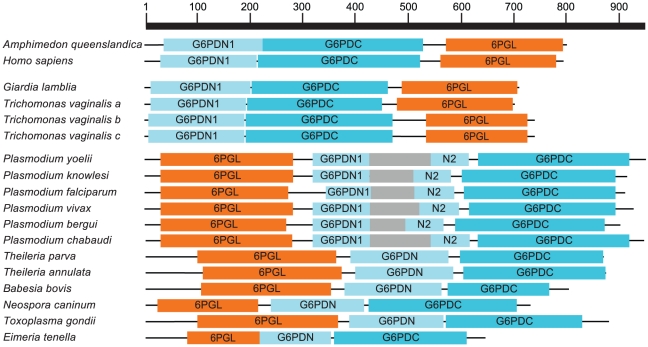
Fusions of the G6PD (N and C terminal regions) and 6PGL genes found in excavates, apicomplexans, and two representative H6PD/6PGL fusions in metazoans. Domains were identified using the Conserved Domain search feature at NCBI [19]. Amino acid length is shown at the top. Note the inverted orientation of the genes in excavates compared to apicomplexans and metazoans. The insert in the *Plasmodium spp.* G6PD domain is shaded. Accession numbers are given in Table S1.

Further extending the searches to all analyzed genomes (Figure 2) revealed additional fusions of 6PGL and G6PD in the excavates *Giardia lamblia*, and *Trichomonas vaginalis*. Interestingly, the order of the fused genes in these species is opposite of that in apicomplexans (Figure 4), suggesting an independent origin for the fusion event in this group. The *T. vaginalis* genome, which is known to contain many examples of gene duplication [17], [31], appears to contain three paralogous copies of the fused gene. We confirmed that the *G. lamblia* gene is indeed fused and not the result of faulty annotation by identifying an EST encoding the fusion protein. Two EST reads (NCBI: EV504738.1 and EV504739.1) derived from opposing ends of the cDNA clone GIARDIA123G04 encode portions of G6PD and 6PGL, respectively. The presence of this EST pair shows that the fusion protein is expressed as a single transcript.

### Origins of the G6PD/6PGL fusion in apicomplexan and excavate genomes

To test which evolutionary scenario is most likely for the emergence of the fusion genes in apicomplexans and excavates we created a phylogenetic tree of G6PD. (The rate of 6PGL sequence evolution is high [32], making it difficult to produce a reliable alignment.) We aligned the sequences of the putative G6PD regions of the apicomplexan and excavate fusion proteins with G6PD sequences from species representing a wide variety of organisms. We then performed a maximum likelihood analysis, with the clade containing bacterial G6PD serving as the outgroup. For species where more than one paralog of G6PD was identified, we included all complete versions encoded in the genome.

**Figure 5:**
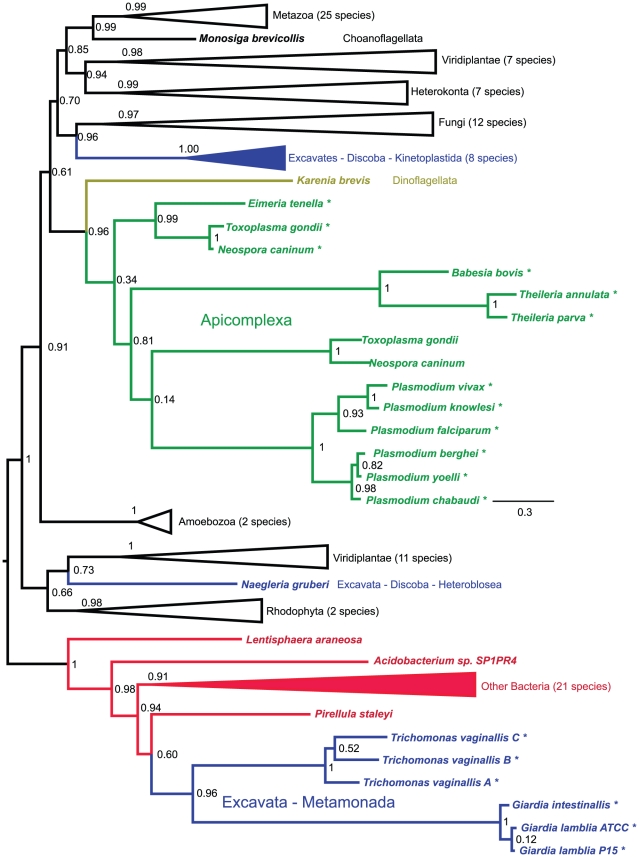
Maximum likelihood tree of G6PD protein sequences. The major eukaryotic groups are collapsed; for a full version of the tree see Figure S1. Bootstrap values are given for selected nodes. Asterisks indicate fused genes. Accession numbers for all sequences used in the tree are given in Table S1.

A number of conclusions about the evolutionary history of these gene fusions can be drawn based on the phylogenetic tree shown in Figure 5. (This figure shows only the position of major eukaryotic groups in the tree; for the full tree, see Figure S1. For a complete list of the organisms in the tree and accession numbers, see Table S1.) First, since the apicomplexan fusion genes branch with a non-fused gene from dinoflagellates, the closest relatives of the apicomplexans, we can assume that the fusion between G6PD and 6PGL occurred after the divergence of apicomplexans and dinoflagellates. Second, the G6PD/6PGL fusion gene is clearly an ancestral feature common to *Trichomonas* and *Giardia*, and we would expect to find this gene in any species that diverged recently from either lineage. Third, the G6PD part of the fusion gene in *Trichomonas* and *Giardia* seems to have been horizontally transferred from a bacteria to an ancestor of both organisms, a fact previously stated in the *Giardia* genome paper [16]. This gene may have been transferred together with a 6PGL, as these genes are commonly found in the same operon in bacteria [33], or they may have been fused prior to the horizontal transfer. If the two genes were not already fused, they became so in a eukaryotic ancestor to both organisms.

### Fusion of H6PD in the animal lineage

**Figure 6:**
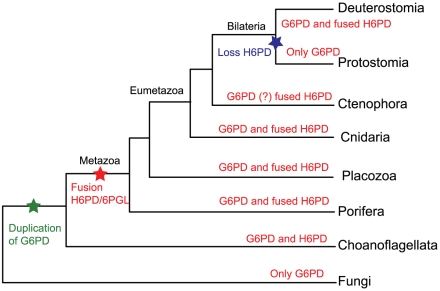
Phylogenetic tree of opisthokonts. Adapted from [34]. The tree shows the duplication of G6PD, leading to the presence of H6PD in an ancestor to metazoa and choanoflagellates (green star), and the subsequent fusion of H6PD to 6PGL in an ancestor of only Metazoa (red star). Protostomes subsequently lost their copy of H6PD (blue star) and Aves lost their copy of G6PD (not shown; see Figure 2).

**Figure 7:**
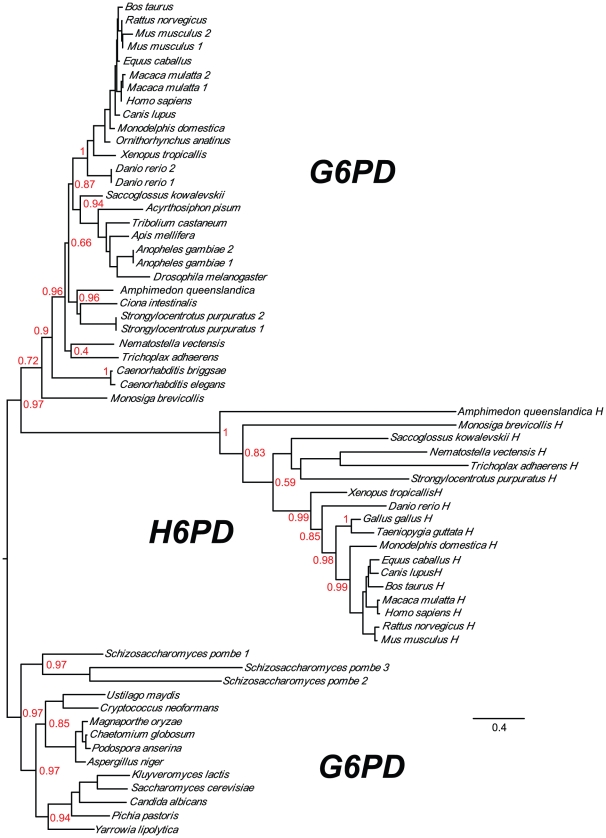
Maximum likelihood tree of Opisthokont G6PD and H6PD proteins. All G6PD sequences are non-fused. Fungi are used as an outgroup for choanoflagellates and metazoans and possess only G6PD orthologs. Both metazoans and choanoflagellates have H6PD genes in addition to G6PD orthologs, both of which were produced by a duplication of G6PD in an ancestor to these groups. All H6PD sequences (labeled with an H following the species name), with the exception of *Monosiga brevicollis*, are fused with 6PGL. Note that in the H6PD subtree the sequences representing the two earliest diverging species, the sponges and choanoflagellate, are switched from the expected order. Accession numbers are given in Table S1.

Orthologs of H6PD were found only in the genomes of metazoans and their most closely related relatives, the choanoflagellates. Fungi contain single, non-fused genes encoding both G6PD and 6PGL. Since the close relationship of animals and fungi is well established, we can conclude that the G6PD gene duplicated in the opisthokont lineage after the divergence of fungi, with one of the genes (H6PD) later becoming fused to 6PGL (Figure 6). The timing of these events can be estimated based on the distribution of the genes in early diverging animals and their closest relatives, the choanoflagellates. Figure 7 shows a phylogenetic tree of the G6PD and H6PD genes of metazoans and one choanoflagellate, using sequences from fungi as the outgroup. Both G6PD and a fused H6PD are present in the sponge *Amphimedon queenslandica*, the earliest animal clade for which data exists [34]. (The G6PD gene in this species is currently miscalled as a fusion to an adjacent TBC superfamily protein.) Thus, both the duplication of G6PD and the fusion of the resulting H6PD with 6PGL must have occurred prior to the divergence of the sponges.

A more distant relative of animals, the choanoflagellate *Monosiga brevicollis* [35], has a non-fused G6PD gene that branches near the base of the metazoan clade in Figure 7. This species also contains what appears to be an H6PD gene based on BLAST analyses, but the gene seems to be fused at the N-terminus to a gene coding a protein containing WD40 repeats rather than to 6PGL. Regardless of whether this fusion is genuine or an artifact, the *Monosiga* H6PD is clearly not fused to its 6PGL, the only copy of which (XP_001745614.1) is present on a different genome scaffold (H6PD is found on MONBRscaffold_9, whereas 6PGL is found on MONBRscaffold_38). The simplest explanation for these data is that the G6PD gene duplicated prior to the divergence of animals and choanoflagellates, with one of the copies undergoing a fusion with 6PGL in animals before the divergence of sponges. H6PD has since been lost from a few clades of animals, as this sequence is not present in the cnidarian *Hydra magnipapillata*, ascidians belonging to the genus *Ciona*, and all species of protostomes. The G6PD gene is missing from the genomes of birds; these genomes have only the fusion H6PD.

### Fusion of G6PD and 6PGD in Phaeodactylum tricornutum

The fusions described here between G6PD and 6PGL are not the only ones observed in the pentose phosphate pathway. The genome of the stramenopile *Phaeodactylum tricornutum* contains two paralogous copies of G6PD (XP_002183714.1 and XP_002185945.1). One of these copies (XP_002183714.1) is fused with the enzyme phosphogluconate dehydrogenase (6PGD). 6PGD catalyzes the step immediately following that of 6PGL, the conversion of 6-phosphogluconate to ribulose-5-phosphate, with the formation of another molecule of NADPH from NADP (Figure 1). Two other copies of 6PGD are present in this genome (XP_002179525.1, XP_002179526.1) and 6PGL is present in one copy (XP_002176734.1). This fusion between G6PD and 6PGD appears to be restricted to this species and its closest relatives, as a BLAST search against the non-redundant (nr) database of NCBI returns no other such fusions. A close inspection of the genomes available at other online sources confirmed that these two genes are separated in the nine other fully sequenced stramenopile genomes. Though still present in GenBank (XP_002185945.1), this fusion has been eliminated and replaced by non-fused copies of G6PD and 6PGD in the most recent annotation of the *P. tricornutum* genome by the Joint Genome Institute, casting doubts on its legitimacy. However, the two genes are separated by only 39 nucleotides, lie in the same reading frame and the first gene lacks a stop codon, suggesting they are transcribed together and translated as a single protein. Because the sequence of the G6PD portion of the gene changed slightly in the new annotation, we used the new, non-fused, version of the gene in our analyses (Table S1).

## Discussion

### The 6PGL/G6PD gene fusion in apicomplexans

The 6PGL/G6PD fusion gene was originally identified in a handful of *Plasmodium* species, and at the time the authors stated that the fusion must have occurred in an ancestor of the *Plasmodium* genus [32]. However, no genomic data were available for many of the species we have analyzed here, and it was not possible to determine how long ago the fusion occurred. The genomes of many protist species, both parasitic and non-parasitic, have been sequenced since then and provide a clearer picture of the distribution of this fusion gene.

Figure 3 shows the currently accepted phylogeny of apicomplexans [36]–[38]. Based on our investigation of species beyond *Plasmodium*, we conclude that the 6PGL/G6PD fusion predates the divergence of all sequenced apicomplexans except for those in the *Cryptosporidium* lineage. The three *Cryptosporidium* species tested in this study lack genes encoding 6PGL and G6PD. Since *Cryptosporidium* holds a key position as one of the earliest branching apicomplexans (Figure 3) [36]–[39], we cannot at this time determine whether the two genes were fused or non-fused before they were lost in this lineage. *Cryptosporidium* has limited biosynthetic capabilities and is thought to import amino acids, nucleotides and simple sugars through several different transporter proteins [40]. By obtaining these metabolites through extracellular transport, the parasite can avoid producing the high levels of reactive oxygen species commonly generated during their metabolism. A lower level of reactive oxygen species may explain how *Cryptosporidium* can survive without the NADPH-producing oxidative branch of the pentose phosphate pathway.

Sequences from dinoflagellates, the closest relatives of the apicomplexans, are poorly sampled, and the lone species for which genome data are publicly available, *Perkinsus marinus* [41], also appears to have lost both 6PGL and G6PD. However, an EST from another dinoflagellate, *Karenia brevis*, contains an open reading frame encoding G6PD (NCBI: CO062433 and CO064937) [42]. This sequence shows no evidence of a 6PGL gene at its 5′ end, which encodes 70 amino acids that share no sequence similarity with previously described proteins, and branches with the apicomplexans in the G6PD phylogenetic tree (Figure 5). Based on the close relationship of the apicomplexan fusion to this non-fused version of G6PD, and considering the rarity with which fused genes can be expected to revert to their initial state [43], it appears that the apicomplexan fusion arose after their divergence from dinoflagellates. The genomes of the ciliates *Tetrahymena thermophila* and *Paramecium tetraurelia*, which represent the earliest branching alveolates, do not contain copies of these genes. These genes were likely lost from ciliates following their divergence from the other alveolates. Non-fused copies of both G6PD and 6PGL are present in the genomes of several stramenopiles, the closest relatives of the alveolates.

Since the genes are not fused in *Karenia brevis*, this event must have occurred in the apicomplexan lineage after its divergence from dinoflagellates. Because both genes were lost in Cryptosporidia, our data do not allow us to pinpoint exactly when during the evolution of apicomplexans the two genes were fused. However, as the fusion is present in all apicomplexan species analyzed here with the exception of Cryptosporidia, it must have happened before the divergence of these species. Thus we place the origin of the 6PGL/G6PD gene fusion after the divergence of dinoflagellates and apicomplexans, but before the divergence of Coccidia, Piroplasmida and Haemosporidia apicomplexans. As data become available for more basal apicomplexans, it may be possible to further narrow this range. For example, now that the gregarines have been identified as a sister group to Cryptosporidia [44], [45], pertinent data may be obtained for this branch of the tree. Efforts are also underway to sequence the genomes of a colpodellid (the earliest diverging apicomplexans), and of *Chromera velia*, a photosynthetic alveolate sister to the apicomplexans. G6PD sequences from these species should be easy to identify and will improve on the estimates we present here.

### A G6PD insertion is limited to Plasmodium spp

A unique feature that stands out in the *Plasmodium* G6PD sequences is the well-studied insertion in the N-terminal domain of the gene and protein (Figure 3; Figure 4). Following its discovery further studies showed that this region of the protein is essential for the function of the enzyme, at least in *P. berghei* [46]. However, it was not clear if this highly variable feature was limited to some or all *Plasmodium* species, or if it was more widespread among the apicomplexans. All the G6PD genes in *Plasmodium* species contained insertions of varying sizes at similar locations in each protein, but this feature was not seen in other apicomplexan species. This insertion therefore appears to be a unique character restricted to, and present in all species of, the genus *Plasmodium*.

### H6PD is a homolog of G6PD present in metazoans and choanoflagellates

It has been known for decades that some metazoans express two different glucose-6-phosphate dehydrogenase enzymes, G6PD and H6PD. The main biological functions recognized for these two enzymes are different. H6PD is localized in the ER lumen where it helps to regulate the oxidative state of glucocorticoid hormones. The NADPH generated by H6PD is used by 11 β-HSD1 to reduce 11-ketoglucocorticoids to their 11 β-hydroxy derivatives [9]. Disruption of G6PD function leads instead to a lack of NADPH in red blood cells, a cell type that does not express H6PD [11]. H6PD has also been shown biochemically and by sequence analysis to possess 6PGL activity at its C-terminus [8], [12] due to a fusion of these two genes, a feature not shared with G6PD.

Before nucleotide or protein sequence comparison methods were widespread, biochemical assays exploiting the broad substrate specificity of H6PD were used to differentiate it from G6PD. These early studies identified two distinct enzymes in several mammals [47], fishes [3], [4], and echinoderms [5]. Based on these findings, it was proposed that H6PD originated from G6PD before the evolution of echinoderms in the early Paleozoic era. These studies also suggested that the enzyme was absent from pigs [47] and from several species of sea urchins [5]. Accordingly, only one version of G6PD can be identified in the genome of the pig *Sus scrofa*, suggesting a recent loss of H6PD, though the presence of both genes in the sea urchin *Strongylocentrotus purpuratus* contradicts these older studies.

Now that full genome sequences are widely available, we were able to search easily for G6PD and H6PD. With the exception of birds, we were able to identify G6PD in all opisthokont species analyzed. H6PD orthologs are found in most classes of metazoans, with the major exception of protostomes. This gene is absent from all fully sequenced protostome genomes, and a BLAST search against all protostome sequences in GenBank returns no orthologs to H6PD. Although the genomes of most metazoans and the sole choanoflagellate available have orthologs of both G6PD and H6PD, the fungal genomes searched contain only G6PD. These data suggest that H6PD originated from a duplication of G6PD in an ancestor of metazoans and choanoflagellates, after these lineages diverged from fungi. Interestingly, the choanoflagellate H6PD is not fused with 6PGL, while those of all metazoans studied, including the sponge *Amphimedon* are fused. This suggests that the H6PD fusion occurred in the metazoan lineage some time after their divergence from choanoflagellates but before the branching of sponges.

### An independent fusion of the G6PD and 6PGL genes occurred in an ancestor of Giardia and Trichomonas

**Figure 8:**
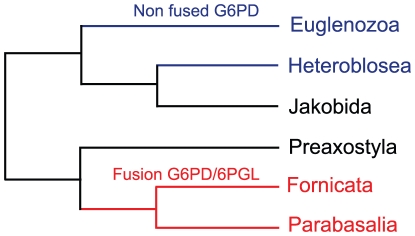
Phylogenetic tree of excavates. Adapted from [28]. The taxa in red contain a fusion between G6PD and 6PGL, while those in blue have non-fused copies of G6PD. The taxa in black have no fully sequenced genome and have no G6PD sequence available for analysis. The three taxa shown at the top form a proposed monophyletic group, Discoba, while the three on the bottom form the monophyletic group Metamonada. Note that the G6PD ortholog in Fornicata (*Giardia*) and that in Parabasalia (*Trichomonas*) appear to be derived from a bacterial gene transfer (see Figure 5).

A closer analysis of a homologous G6PD gene in *Giardia lamblia* led to the discovery that this gene is fused with 6PGL in this species, but in the opposite orientation from the apicomplexan genes. Subsequent searching for fusion genes encoding proteins where G6PD and 6PGL are found at the N-terminus and C-terminus, respectively, detected three paralogous genes in *Trichomonas vaginalis*. We did not find any other fusions among the excavates, a group that includes *Giardia*, *Trichomonas*, and assorted other groups including the kinetoplastid parasites *Trypanosoma* and *Leishmania*. These and other kinetoplastids express the two genes separately, showing that the fusion must have occurred within Excavata. Because excavate phylogenies are difficult to determine due to long-branch attraction of the rapidly evolving species in this group [48], it is not a simple task to determine which species are likely to have this fusion. Figure 8 shows the currently accepted relationships among selected excavates [28]. *Giardia* is known to belong to a monophyletic group called the Fornicata. Since *Trichomonas*, a parabasalid, branched prior to the emergence of this group, we can state that all fornicata and parabasalid species are expected to contain the G6PD/6PGL fusion gene, since the fusion predated their divergence. Identification of the G6PD and 6PGL genes in sister groups to these clades will allow a more accurate age estimate for this fusion. Interestingly, the G6PD gene seen in *Giardia* and *Trichomonas* seems to have been horizontally transferred from a prokaryote, as opposed to the G6PD homologs in other excavates that group with eukaryotes in the tree (Figure 5). Whether this gene was fused or not at the time of its transfer is difficult to determine because the phylogenetic trees we have produced for 6PGL have been inconclusive.

### Fusion genes as identifiers for monophyletic groups within alveolates and excavates

The two genes identified here have the potential to delineate sharp phylogenetic boundaries within two particularly intractable clades. A series of fusion genes have been useful in defining deep branches in the larger tree of eukaryotes [43], and a similar approach may be possible with the current G6PD/6PGL fusions. The deepest branches in the alveolates, and within the apicomplexans, are currently disputed. The monophyly of dinoflagellates and apicomplexans, to the exclusion of the ciliates, has received the majority of the support in recent studies. Since we have identified a dinoflagellate transcript that encodes only G6PD and not 6PGL, we doubt it will be possible to support or reject this grouping by searching for fusions of these two genes. The earliest branches in the apicomplexan lineage are also in question, and may be clarified as more species are sequenced. For example, if a 6PGL/G6PD fusion with an insert in the N-terminal region of G6PD were found in a gregarine species, it would provide a compelling argument for a recently proposed relationship between the plasmodia and gregarines [49].

Excavates are a complex group of eukaryotes and their monophyly is still debated [27]. The G6PD/6PGL fusion supports the monophyly of the Parabasalia (*Trichomonas*) and Fornicata (*Giardia*), which together with Preaxostyla (no genome available) form the phylum Metamonada, to the exclusion of other excavates like Euglenozoa (*Trypanosoma*, *Leishmania*) and Heterolobosea (*Naegleria*), which together with Jakobids (no genome available) form the phylum Discoba (Figure 8). Considering that the fusion seen in excavates also appears to be horizontally transferred from a prokaryote, this gene should be an extremely useful characteristic when grouping species within Metamonada, since both the fusion and a phylogenetic analysis can confirm placement within this clade.

### Functional significance of the G6PD/6PGL fusion genes

The independent fusion of the G6PD and 6PGL genes three times during evolution suggests a possible selective benefit to linking these two proteins. Some fused proteins are known to increase the overall rate of the reaction catalyzed by the constituent domains, due to a phenomenon known as substrate channeling [50]. If the active sites of two enzymes are held in proximity, it is possible for the products of the first reaction to be fed immediately to the active site of the next enzyme. This increases the association rate of the second enzyme and prevents other enzymes from diverting the first product, speeding that part of the reaction. It is therefore possible that the organisms containing fusions between G6PD and 6PGL have an evolutionary advantage due to a more efficient oxidative branch of the pentose phosphate pathway. It must be noted however that, while an increased reaction rate may have been beneficial to a distant ancestor of these extant organisms, the advantage may have disappeared over time, leaving these fusions as relics. Gene fission events in which two fused genes revert to their former state are rare, requiring that the gene somehow obtain an additional promoter, terminator, start codon, and stop codon [51]. The fusions may also have played no role in the fitness of these organisms, reflecting instead a neutral change following a deletion or translocation event. Additional experiments, including the biochemical determination of reaction rates and resolution of the three-dimensional structure of the protein, may reveal whether these fusions are indeed adaptive.

## Supporting Information

Figure S1 **Complete version of the maximum likelihood tree of G6PD protein sequences shown in** **Figure 5** **.** Bootstrap values are given in the tree nodes. Red indicates fused genes. A key for the abbreviations used to represent the sequences in the tree is given in Table S1.
(EPS)

Table S1 **List of all sequences used in this study.** This file contains the list of all species analyzed together with the accession number and data source for each sequence.
(XLSX)
